# Induced Pluripotent Stem Cell Therapy Ameliorates Hyperoxia-Augmented Ventilator-Induced Lung Injury through Suppressing the Src Pathway

**DOI:** 10.1371/journal.pone.0109953

**Published:** 2014-10-13

**Authors:** Yung-Yang Liu, Li-Fu Li, Jui-Ying Fu, Kuo-Chin Kao, Chung-Chi Huang, Yueh Chien, Yi-Wen Liao, Shih-Hwa Chiou, Yuh-Lih Chang

**Affiliations:** 1 Chest Department, Taipei Veterans General Hospital, Taipei, Taiwan; 2 Institute of Clinical Medicine, School of Medicine, National Yang-Ming University, Taipei, Taiwan; 3 Department of Internal Medicine, Division of Pulmonary and Critical Care Medicine, Chang Gung Memorial Hospital and Chang Gung University, Taoyuan, Taiwan; 4 Department of Respiratory Therapy, Chang Gung Memorial Hospital, Taoyuan, Taiwan; 5 Department of Medical Research & Education, Taipei Veterans General Hospital, Taipei, Taiwan; 6 Institute of Pharmacology, School of Medicine, National Yang-Ming University, Taipei, Taiwan; University of Illinois College of Medicine, United States of America

## Abstract

**Background:**

High tidal volume (V_T_) mechanical ventilation (MV) can induce the recruitment of neutrophils, release of inflammatory cytokines and free radicals, and disruption of alveolar epithelial and endothelial barriers. It is proposed to be the triggering factor that initiates ventilator-induced lung injury (VILI) and concomitant hyperoxia further aggravates the progression of VILI. The Src protein tyrosine kinase (PTK) family is one of the most critical families to intracellular signal transduction related to acute inflammatory responses. The anti-inflammatory abilities of induced pluripotent stem cells (iPSCs) have been shown to improve acute lung injuries (ALIs); however, the mechanisms regulating the interactions between MV, hyperoxia, and iPSCs have not been fully elucidated. In this study, we hypothesize that Src PTK plays a critical role in the regulation of oxidants and inflammation-induced VILI during hyperoxia. iPSC therapy can ameliorate acute hyperoxic VILI by suppressing the Src pathway.

**Methods:**

Male C57BL/6 mice, either wild-type or Src-deficient, aged between 2 and 3 months were exposed to high V_T_ (30 mL/kg) ventilation with or without hyperoxia for 1 to 4 h after the administration of Oct4/Sox2/Parp1 iPSCs at a dose of 5×10^7^ cells/kg of mouse. Nonventilated mice were used for the control groups.

**Results:**

High V_T_ ventilation during hyperoxia further aggravated VILI, as demonstrated by the increases in microvascular permeability, neutrophil infiltration, macrophage inflammatory protein-2 (MIP-2) and plasminogen activator inhibitor-1 (PAI-1) production, Src activation, nicotinamide adenine dinucleotide phosphate (NADPH) oxidase activity, and malaldehyde (MDA) level. Administering iPSCs attenuated ALI induced by MV during hyperoxia, which benefited from the suppression of Src activation, oxidative stress, acute inflammation, and apoptosis, as indicated by the Src-deficient mice.

**Conclusion:**

The data suggest that iPSC-based therapy is capable of partially suppressing acute inflammatory and oxidant responses that occur during hyperoxia-augmented VILI through the inhibition of Src-dependent signaling pathway.

## Introduction

Acute respiratory distress syndrome (ARDS) is characterized by pulmonary edema, increased alveolocapillary permeability, leukocyte infiltration, and the release of cytokines (because of severe epithelial and endothelial injury) [1]–[3]. The management of ARDS often necessitates the use of mechanical ventilation (MV) with high levels of oxygen, especially in the first few hours after intubation. This allows for adequate maintenance of the oxygenation of vital organs. However, both hyperoxia and high-stretch MV can damage normal lung tissue [4]–[14]. Hyperoxia may cause neutrophil infiltration and pulmonary edema [8], [13]. Even after as little as 3 h of hyperoxia, previous studies have shown that the gene expression of tumor necrosis factor-α (TNF-α) in alveolar macrophages and alveolar epithelial cells can be amplified [15], [16]. Pathologic lung over-distension may occur in the remaining normal lung in patients with ARDS, even when using a low tidal volume (V_T_) strategy. Thus, over-distention of lung tissue during MV was identified as the triggering factor that initiated ventilator-induced lung injury (VILI). Concomitant hyperoxia further aggravated the progression of VILI and led to increased production of murine macrophage inflammatory protein-2 (MIP-2), and blocking MIP-2 reduced the occurrence of lung injury in an animal model [17], [18]. We previously demonstrated that hyperoxia augmented VILI through the activation of plasminogen activator inhibitor-1 (PAI-1), which was mediated by redox-sensitizing transcription factor nuclear factor-kappaB (NF-κB) in mice [19]. In clinical practice, excessive oxygen supplementation used in mechanically ventilated patients with acute lung injury (ALI) was associated with deteriorating lung function and pulmonary outcomes [14].

Oxidative stress seems to play a pivotal role in the inflammatory process that occurs during VILI concomitant with hyperoxia [20]. Cyclic mechanical stretch of the lung epithelium is involved in the VILI inflammatory process through the excessive production of reactive oxygen species (ROS) by activating nicotinamide adenine dinucleotide phosphate (NADPH) oxidase *in* *vitro* [21]. At the cellular level, excessive alveolar stretch during high V_T_ ventilation could activate a series of intracellular signaling pathways, most of which are regulated by protein phosphorylation. The Src protein tyrosine kinase (PTK) family is one of the most critical families to intracellular signal transduction that is related to acute inflammatory responses [22]–[23]. Src PTKs mediate the tyrosine phosphorylation of p47^phox^ in hyperoxia-induced activation of NADPH oxidase and ROS production in human pulmonary arterial endothelial cells [24]. Chang et al. demonstrated that intratracheal administration of umbilical cord blood-derived mesenchymal stem cells (MSCs) attenuated hyperoxia-induced lung injury in neonatal rats by suppressing both cytosolic and membrane p47^phox^, but the actual molecular mechanism behind this phenomenon was not shown [25].

Recent studies have demonstrated that induced pluripotent stem cells (iPSCs) can be generated from mouse embryonic fibroblasts and from adult human fibroblasts through ectopic expression of defined transcription factors, Oct3/4, Sox2, c-Myc, and Klf4 [26]–[29]. Embryonic stem cells (ESCs) and iPSCs exhibit similar morphology, proliferative abilities, surface antigens, gene expression, epigenetic status of pluripotent cell-specific genes, and telomerase activity [27], [28]. As well as their self-renewal capacity and ability to differentiate into three germ layers, iPSCs can be derived from the somatic cells of a patient. This method avoids the ethical considerations and the possibility of immune rejection after transplantation that are associated with ESCs [27]–[29]. Remarkably, patient-specific pulmonary alveolar proteinosis iPSCs were shown to recapitulate the cellular phenotype of this hereditary disorder and represent an *in* *vitro* disease model for studies of disease pathophysiology or for drug testing [30]. Dr. Masayo Takahashi has been conducting a pilot clinical study on autologous iPSC therapy and age-related macular degeneration since August 1, 2013 [31]. Therefore, iPSCs are regarded as a suitable candidate for disease modeling, gene therapy, or cell replacement used for autologous transplantation without the risk of rejection or ethical concerns. Nevertheless, the risk of tumorigenicity of iPSCs is still in doubt. A recent study of a mouse model of lipopolysaccharide (LPS)-induced ALI demonstrated that iPSCs can exert anti-inflammatory effects [32]. Chimenti et al. indicated that pretreatment with MSCs reduced VILI in rats subjected to high V_T_ ventilation, but the exact mechanisms underlying this phenomenon were not described [33]. Moreover, the roles of iPSC therapy in hyperoxia-augmented VILI have not been fully delineated and require further exploration.

In this mouse model of hyperoxia-augmented VILI, we examine the relationships among high V_T_ ventilation and hyperoxia, iPSCs, MIP-2 and PAI-1 production, intracellular oxidative stress, and activation of Src and NADPH oxidase signaling using Src knock-out mice. We hypothesized that intravenous injection of Oct4/Sox2/Poly(ADP-ribose) polymerase 1 (Parp1) (OSP)-iPSCs would decrease neutrophil infiltration, oxidative stress, lung edema, and MIP-2 and PAI-1 production in mice exposed to high V_T_ ventilation with hyperoxia through the Src pathway.

## Results

### Mouse iPSCs generated from OSP-overexpressing MEFs

**Figure 1:**
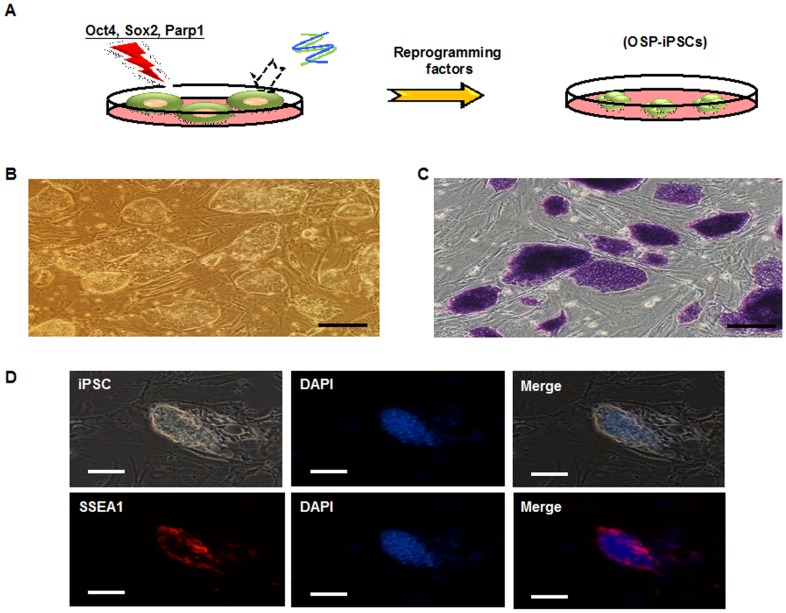
Characterization of Oct4/Sox2/Parp1(OSP)-reprogrammed iPSCs. (A) Parp1 is able to replace Klf-4 or c-Myc to generate mouse OSP-iPSCs cotransfected with Oct-4 and Sox-2. (B) Morphology of OSP-iPS cell colonies. (C) OSP-iPSC colonies were strongly positive for alkaline phosphatase stain. (D) The high passages of OSP-iPSCs were positive for SSEA-1 by immunofluorescent staining. Scale bars represent 200 µm (B & C) and 100 µm (D). DAPI = 4′, 6-diamidino-2-phenylindole; iPSC = induced pluripotent stem cell; OSP-iPSC = Oct4/Soc2/Parp1-reprogrammed induced pluripotent stem cell; SSEA-1 = stage-specific embryonic antigen 1.

The proto-oncogene, c-Myc, is an essential factor for enhancing reprogramming efficiency, but it also increases the risk of tumorigenicity of the reprogrammed somatic cells [34]. Chiou et al. demonstrated the potential of Parp1 for replacing Klf4 and c-Myc. They also indicated that Parp1 cotransfected with Oct-4 and Sox-2 (OSP; Figure 1A) in mouse embryonic fibroblasts (MEFs) could effectively generate iPSC lines (OSP-iPSCs; Figure 1B) [35]. The high passages of OSP-reprogrammed iPSCs were positive for markers of mouse ESCs, such as alkaline phosphatase (ALP) activity (Figure 1C) and stage-specific embryonic antigen 1 (SSEA-1; Figure 1D). Six weeks after transplantation of these iPSCs into the dorsal flanks of nude mice, the formation of teratomas that contained various tissues was observed (data not shown). The data indicated that OSP-reprogrammed iPSCs present a high pluripotency potential, which shared significant similarity to iPSCs reprogrammed from MEFs cotransfected with Oct-4, Sox2, Klf-4, and c-Myc [35].

### Inhibition of the effects of hyperoxia on lung stretch-induced Src activation by iPSCs

**Figure 2:**
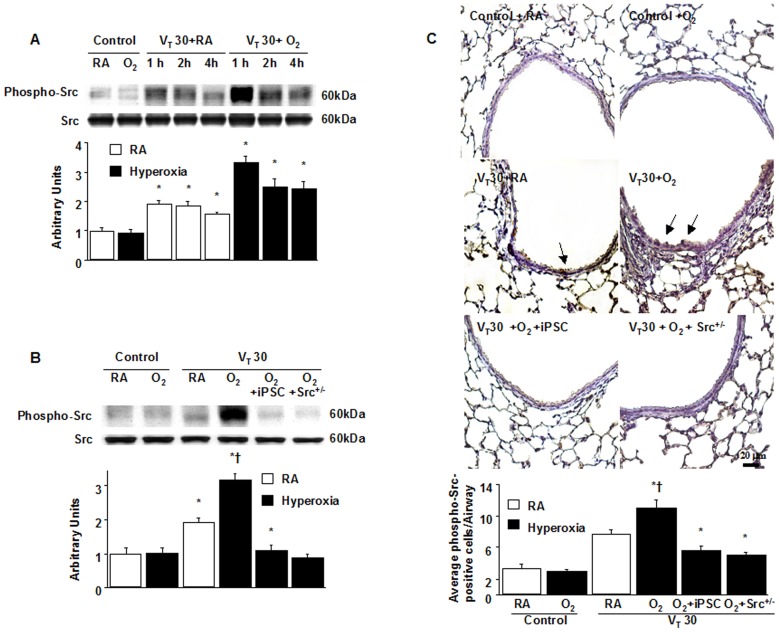
iPSCs and Src-deficient mice suppressed hyperoxia-augmented lung stretch-induced Src phosphorylation. (A, B) Western blot was performed using an antibody that recognizes the phosphorylated Src expression and an antibody that recognizes total Src expression from the lungs of nonventilated control mice and those subjected to V_T_ 30 ml/kg (V_T_ 30) with room air or hyperoxia at indicated time periods. Arbitrary units were expressed as the ratio of phospho-Src to Src (n = 5 per group). (C) Representative micrographs (x400) with phosphorylated Src staining of paraffin lung sections and quantification were from the lungs of nonventilated control mice and those subjected to V_T_ at 30 ml/kg for 4 h with room air or hyperoxia (n = 5 per group). iPSCs (5×10^7^ cells/kg, suspended in PBS) were injected via tail vein 1 h before mechanical ventilation. A dark-brown diaminobenzidine signal identified by arrows indicates positive staining for phospho-Src in the lung epithelium or interstitial, whereas shades of bluish tan signify nonreactive cells. *P<0.05 versus the nonventilated control mice with room air; **†**P<0.05 versus all other groups. Scale bars represent 20 µm. iPSC = induced pluripotent stem cell; O_2_ = mice with hyperoxia; PBS = phosphate-buffered saline; RA = mice with room air; Src^+/−^ = Src deficient mice.

MSCs attenuated hyperoxia-induced lung injury in neonatal rats [25]. We employed high V_T_ (30 mL/kg) ventilation with room air or hyperoxia for 1 to 4 h to induce VILI in mice and examined the treatment effects of intravenously delivered iPSCs. The physiological conditions at the beginning and end of ventilation are shown in Table 1. The normovolemic statuses of mice were maintained by monitoring their mean artery pressure. We measured Src phosphorylation in mice subjected to a V_T_ of 30 mL/kg to investigate the role of the Src pathway in this VILI model and determine the effects of hyperoxia on stretch-induced Src activation (Figures 2A, 2B). Time-dependent increases in the phosphorylation of Src occurred, but the expression of total nonphosphorylated proteins of Src did not change significantly. The activation of Src increased after 1 h of ventilation with a V_T_ of 30 mL/kg and remained elevated after 4 h of MV compared with those of the nonventilated control mice. Administering hyperoxia induction increased the significance of Src phosphorylation in mice after a V_T_ of 30 mL/kg. Inhibition of Src by using iPSCs eliminated the V_T_30-induced Src activation during hyperoxia. Consistent with the Western blot results, the positive immunohistochemical staining for Src in the lung epithelium of mice subjected to a V_T_ of 30 mL/kg with hyperoxia was significantly attenuated by the iPSC treatment (Figure 2C).

**Table 1:** Physiologic conditions at the beginning and end of ventilation.

	Control, wild-type,	Control, wild-type,	V_T_ 30 ml/kg, wild-type,	V_T_ 30 ml/kg, wild-type,	V_T_ 30 ml/kg, wild-type,	V_T_ 30 ml/kg, wild-type,
	Room air	Hyperoxia	Room air	Hyperoxia	Hyperoxia with iPSCs	Hyperoxia with Src^+/−^
PH	7.41±0.05	7.38±0.02	7.36±0.04	7.35±0.03	7.36±0.04	7.37±0.02
PaO2 (mmHg)	97.5±0.6	427.5±5.1	86.8±0.7*	386.7±3.8*†	405.7±4.2*	412.6±3.9*
PaCO2 (mmHg)	39.4±0.3	39.6±0.5	36.5±1.2	43.4±1.3	41.6±1.5	41.4±1.8
MAP (mmHg)						
Start	85.7±1.3	86.1±1.8	85.1±1.6	84.7±2.3	84.9±2.6	84.8±2.0
End	85.2±0.8	85.2±0.9	77.8±2.8*	75.3±2.7*†	78.3±1.9*	79.6±2.5*
PIP, mm Hg						
Start			24.2±1.3	25.1±1.2	24.9±1.4	25.0±1.1
End			27.5±1.1	28.6±1.5	28.1±1.2	27.9±1.4

At the end of the study period, we obtained data of arterial blood gases and mean arterial pressure from the nonventilated control mice and mice subjected to V_T_ at 30 mL/kg for 4 h (n = 10 per group). We maintained the normovolemic statuses of mice by monitoring the mean artery pressure. Data are presented as means ± SDs. *indicates that P<0.05 when compared to the nonventilated control mice with room air and **†**indicates that P<0.05 when compared to all other groups. iPSC = induced pluripotent stem cell; MAP = mean arterial pressure; PIP = peak inspiratory pressure; Src^+/−^ = Src-deficient mice; V_T_ = tidal volume. The physiological data on the nonventilated control groups were similar during the experiment and were used as ventilation start data.

### Suppressing the synergistic effects of hyperoxia on high V_T_ ventilation-induced NOX2 expression, oxygen radicals, and inflammatory responses by iPSCs

**Figure 3:**
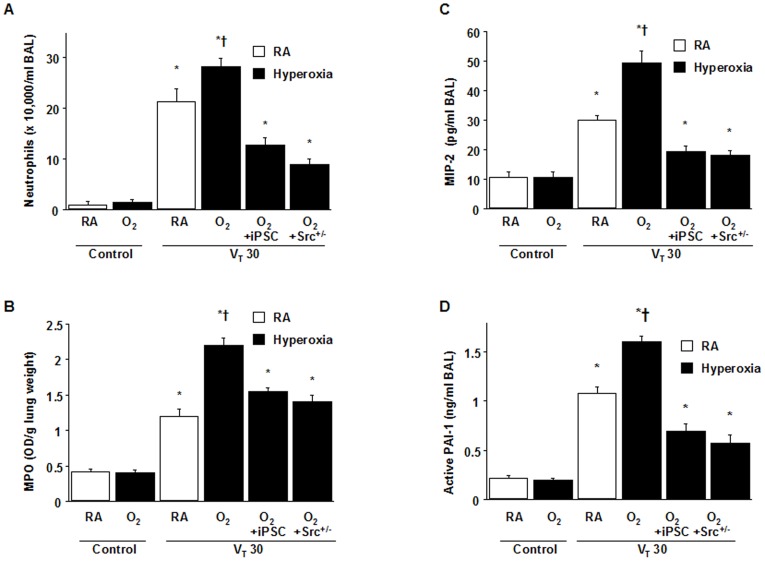
iPSCs and Src-deficient mice attenuated hyperoxia-augmented lung stretch-induced neutrophil sequestration, MIP-2 and PAI-1 production. The effects of administering iPSCs or Src heterozygous knockout on (A) neutrophil infiltration, (B) MPO activity, (C) MIP-2, and (D) PAI-1 secretion in BAL fluid were from the lungs of nonventilated control mice and those subjected to V_T_ at 30 ml/kg for 4 h with room air or hyperoxia (n = 5 per group). iPSCs (5×10^7^ cells/kg, suspended in PBS) were injected via tail vein 1 h before mechanical ventilation. *P<0.05 versus the nonventilated control mice with room air; †P<0.05 versus all other groups. BAL = bronchoalveolar lavage fluid; MIP-2 = macrophage inflammatory protein-2; MPO = myeloperoxidase; PAI-1 = plasminogen activator inhibitor-1.

Neutrophils are the main inflammatory cells involved in the ALI process [2]. We measured neutrophil counts, myeloperoxidase (MPO) activity, and MIP-2 and PAI-1 protein production to determine the effects of hyperoxia on neutrophils, which are a primary source of ROS marginated in the vasculature, lung parenchyma, and alveoli, and to determine the effects of inflammatory cytokines on neutrophils (Figure 3). The results showed increased neutrophil migration into the injured lung and elevated levels of MIP-2 and PAI-1 in mice subjected to a V_T_ of 30 mL/kg with hyperoxia compared with those subjected to V_T_ at 30 ml/kg with room air and nonventilated control mice.

**Figure 4:**
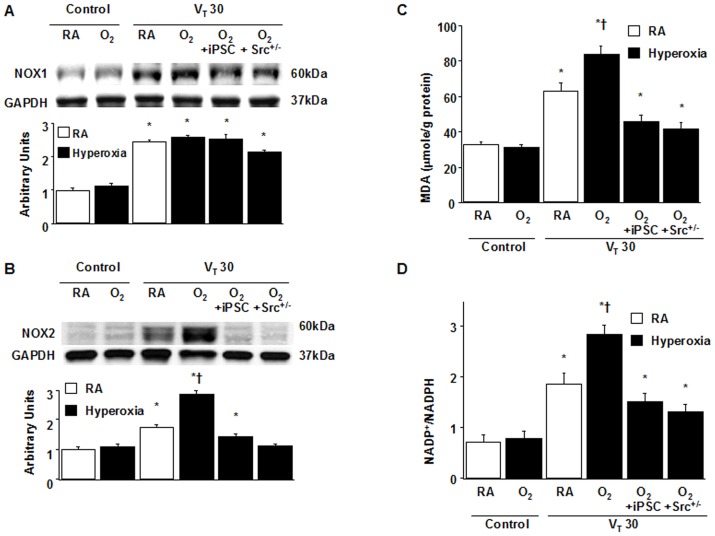
iPSCs and Src-deficient mice abrogated hyperoxia-augmented lung stretch-induced oxidative stress. (A, B) Western blot was performed using antibodies that recognize NOX1 or NOX2 expression and an antibody that recognizes GAPDH expression from the lungs of nonventilated control mice and those subjected to V_T_ 30 ml/kg for 1 h with room air or hyperoxia. Arbitrary units were expressed as the ratio of NOX1 to GAPDH or NOX2 to GAPDH (n = 5 per group). (C) MDA level and (D) NADP^+^-to-NADPH ratio were from the lungs of nonventilated control mice and those subjected to V_T_ at 30 ml/kg for 4 h with room air or hyperoxia (n = 5 per group). iPSCs (5×10^7^ cells/kg, suspended in PBS) were injected via tail vein 1 h before mechanical ventilation. *P<0.05 versus the nonventilated control mice with room air; **†**P<0.05 versus all other groups. GAPDH = glyceraldehydes-phosphate dehydrogenase; MDA = malondialdehyde; NADP^+^ = nicotinamide adenine dinucleotide phosphate; NADPH = reduced NADP^+^; NOX1 = NADPH oxidase 1; NOX2 = NADPH oxidase 2.

Moreover, the upregulation of a crucial oxidant-generating enzyme NADPH oxidase (NOX) 2, and the elevation of critical markers of oxidative stress (the MDA level and NADP^+^-to-NADPH ratio) were demonstrated in mice subjected to a V_T_ of 30 mL/kg with hyperoxia compared with those subjected to a V_T_ of 30 mL/kg with room air and the control mice (Figure 4). No substantial differences on NOX1 expression occurred between mice subjected to a V_T_ at 30 mL/kg with or without hyperoxia (Fig. 4A). These data suggested that an increase in oxidative stress and the upregulation of chemokines for neutrophils were involved in hyperoxia-induced ALI. Remarkably, iPSCs ameliorated levels of NOX2 and MDA, the NADP^+^-to-NADPH ratio, neutrophil infiltration, and MIP-2 and PAI-1 protein elevation (Figures 3 and 4).

### Reduction of the detrimental effects of hyperoxia on high V_T_ -induced VILI by iPSCs

**Figure 5:**
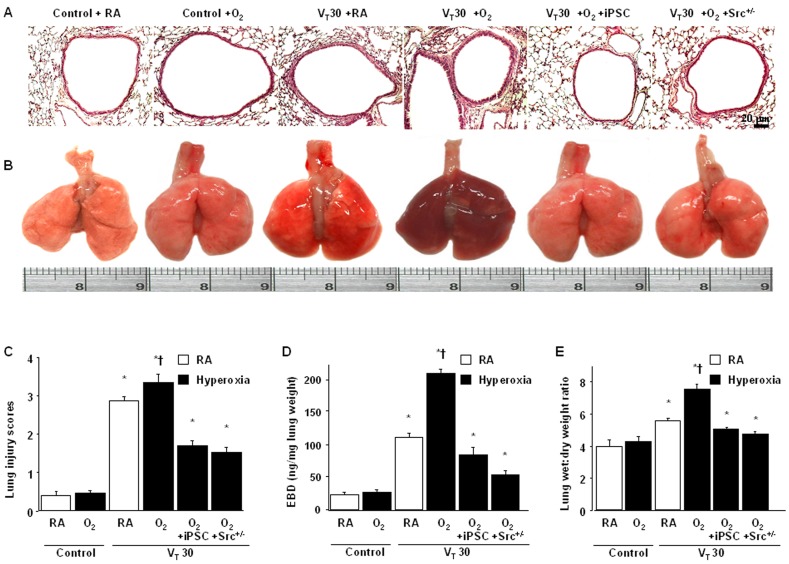
iPSCs and Src-deficient mice reduced hyperoxia-augmented lung stretch-induced lung damage, microvascular leak, and lung edema. (A) Histological examination (x200), (B) gross pathologic findings, (C) lung injury scores, (D) lung EBD, and (E) the wet-to-dry ratio were from the lungs of nonventilated control mice and those subjected to V_T_ at 30 ml/kg for 4 h with room air or hyperoxia (n = 5 per group). iPSCs (5×10^7^ cells/kg, suspended in PBS) were injected via tail vein 1 h before mechanical ventilation. *P<0.05 versus the nonventilated control mice with room air; †P<0.05 versus all other groups. EBD = Evans blue dye.

Histological examinations and the gross pathologic results indicated that the animal lungs injured by MV at a V_T_ of 30 mL/kg with hyperoxia displayed a hemorrhaging pattern, severe congestion, and enlargement (Figures 5A, 5B). The lung injury score quantification confirmed that V_T_30 induced severe lung damage during hyperoxia (Figure 5C). Moreover, we measured the lung Evans blue dye (EBD) and the wet-to-dry weight ratio to determine the effects of high V_T_ ventilation with and without hyperoxia on changes in microvascular permeability and lung water content in VILI (Figures 5D, 5E). The lung congestion and elevation of capillary permeability induced by a V_T_ of 30 mL/kg with hyperoxia were not affected by MEF treatment, but were substantially suppressed by the iPSC treatment (Figure 5). There were statistically significant differences among lung injury score, EBD and wet-to-dry ratio in wild-type or Src-deficient mice with iPSCs treatment and ventilated at V_T_ 30/kg with hyperoxia (the lung injury score quantification: V_T_ 30 ml/kg wild-type mice with iPSCs breathing hyperoxia = 1.7±0.2 versus V_T_ 30 ml/kg Src-deficient mice with iPSCs breathing hyperoxia = 1.4±0.3, P = 0.04; the levels of EBD: V_T_ 30 ml/kg wild-type mice with iPSCs breathing hyperoxia = 69.5±1.8 ng/mg lung weight versus V_T_ 30 ml/kg Src-deficient mice with iPSCs breathing hyperoxia = 56.7±4.1 ng/mg lung weight, P = 0.002; wet-to-dry weight ratio: V_T_ 30 ml/kg wild-type mice with iPSCs breathing hyperoxia = 5.0±0.4 versus V_T_ 30 ml/kg Src-deficient mice with iPSCs breathing hyperoxia = 4.4±0.3, P = 0.03). This data suggested that iPSCs can improve microvascular leakage, lung edema, and total lung injury in a mouse VILI model subjected to a V_T_ of 30 mL/kg with hyperoxia.

### Src-deficient mice reduced the effects of hyperoxia on ventilation-induced microvascular leaks, Src activation, neutrophil sequestration, oxygen radicals, and MIP-2 and PAI-1 production

We investigated whether the beneficial effects provided by iPSCs were mediated through the Src pathway. We used Src-deficient mice to determine the role of Src activation in hyperoxia-augmented VILI. The additive effects of hyperoxia increased microvascular leaks, lung edema, neutrophil influx, MPO levels, NOX2 expression, oxidative stress, Src activation, and MIP-2 and PAI-1 production in mice subjected to a V_T_ of 30 mL/kg. These deleterious changes were substantially attenuated by iPSC treatment and in Src-deficient mice (Figures 2 to 5). The results indicated that iPSCs can suppress high V_T_ ventilation and concomitant hyperoxia-induced oxidative burst and inflammatory responses through inhibiting the Src pathway.

### Reduced hyperoxia-augmented alveolar stretch-induced epithelial apoptosis and improved oxygenation by iPSCs and in Src-deficient mice

**Figure 6:**
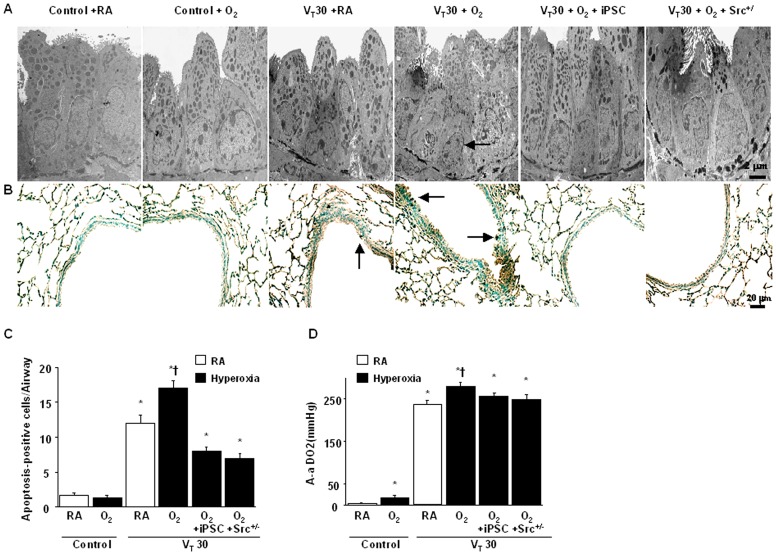
iPSCs and Src-deficient mice abrogated hyperoxia-augmented lung stretch-induced epithelial apoptosis and gas exchange. Representative micrographs with (A) a transmission electron microscopic image (x6000), (B) TUNEL staining of paraffin section (x400), (C) quantitation, and (D) gas exchange (A, n = 3 per group; B, C, and D, n = 5 per group) were from the lungs of the control mice and those subjected to V_T_ at 30 ml/kg for 4 h with room air or hyperoxia. iPSCs (5×10^7^ cells/kg, suspended in PBS) were injected via tail vein 1 h before mechanical ventilation. Highly condensed and fragmented heterochromatin of bronchial epithelial cells indicates apoptosis. A dark-brown diaminobenzidine signal indicated positive staining of apoptotic cells, whereas shades of blue–green to greenish tan signified nonreactive cells. Apoptotic cells are identified by arrows. *P<0.05 versus the nonventilated control mice with room air; †P<0.05 versus all other groups. Scale bars represent 2 or 20 µm. A-aDO_2_ = alveolar-arterial oxygen difference; TUNEL = terminal deoxynucleotidyl transferase-mediated dUTP-biotin nick end-labeling.

Because upregulating Src has been associated with stretch-induced pathway-driven lung inflammation with hyperoxia, we performed transmission electron microscopy (TEM) and terminal deoxynucleotidyl transferase-mediated dUTP nick end-labeling (TUNEL) staining to determine the effects of Src deficiency in mice on high V_T_ ventilation-induced apoptosis of airway epithelial cells (Figures 6A, 6B, 6C). Epithelial apoptosis was confirmed by the characteristic nuclear condensation and cell shrinkage of bronchial epithelium in mice subjected to a V_T_ of 30 mL/kg with hyperoxia compared with those subjected to a V_T_ of 30 mL/kg with room air and the control mice. The increase in V_T_30-induced epithelial apoptosis with hyperoxia decreased by administering iPSCs and in Src-deficient mice. Furthermore, iPSCs and Src heterozygous knockout improved the increase in the gas exchange (alveolar-arterial oxygen difference; A-aDO2) in mice receiving a V_T_ of 30 mL/kg with hyperoxia (Fig. 6D).

## Discussion

High V_T_ ventilation in healthy mice has been used to simulate the small volume of the healthier lung which received most of the ventilation in ARDS. Previous studies demonstrated that hyperexpansion of the lung was the mechanism of volutrauma and biotrauma in VILI [3]–[6]. Although lung-protective ventilation therapy is advantageous, the mortality of ARDS has remained high [36]. In medical practice, high levels of oxygen, especially in the first few hours after intubation, are required to treat patients with ARDS. Hyperoxia has been shown to cause lung edema, destruction of alveolar epithelial barrier, hyaline membrane formation, and interstitial fibrosis [13]. Therefore, the deleterious effect of hyperoxia on VILI should be seriously evaluated to advance the therapy of ARDS. Notably, novel therapies including cell-based therapy are needed to further reduce morbidity and mortality from ARDS. In our previous study, we observed the salutary effects of iPSCs on the LPS-induced ALI in mice [32]. In this mouse acute hyperoxic VILI model, we have demonstrated that high V_T_ ventilation plus hyperoxia further worsened lung damage. Specifically, there were increases in lung edema, microvascular permeability, neutrophil infiltration, production of MIP-2 and PAI-1 of BAL fluid, intracellular oxidative stress and NADPH oxidase activity, increased epithelial apoptosis, and total lung injury via Src activation. Importantly, iPSC therapy could protect mice against high stretch ventilation-induced lung injury concomitant with hyperoxia and restore the functional gas exchange by improving oxygenation. Taken together, we are the first to explore the roles of Src and NADPH oxidase in mediating the beneficial effects provided by iPSCs in hyperoxia-augmented VILI. In addition, we demonstrated that iPSC-based therapy can ameliorate hyperoxia-augmented high V_T_-induced ALI by suppressing oxidative stress, inflammation, and apoptosis through Src-dependent signaling pathway.

Src protein tyrosine kinase (PTK) family is categorized into nonreceptor tyrosine kinases and is one of the most pivotal families for intracellular signal transduction relevant to cell proliferation, migration, differentiation, and apoptotic cell death [22], [23]. It is known that Src PTK is ubiquitously expressed by macrophages, monocytes, neutrophils, alveolar epithelial cells, endothelial cells, and fibroblasts in the lung [22]. Src PTK has been identified as a critical regulator for the recruitment of leukocytes, NADPH oxidase activation and ROS production, and may further upregulate phosphoinositide 3-kinase (PI3K)/serine/threonine-protein kinase B (Akt)/NF-κB pathway in macrophages or alveolar epithelial cells in acute pulmonary inflammation [22], [23]. Recently, mechanical stretch-induced activation of Src was proven to increase lung vascular permeability in mice exposed to MV [37]. A previous study of injurious ventilation applied in isolated perfused murine lung showed that MV increased vascular permeability and pulmonary edema via activation of Src PTK [37]. Moreover, MV can activate Src phosphorylation by activation of adherens junctions, Ca^2+^ entry through stretch-activated cation channels, deformation of cytoskeleton and integrins, focal adhesion kinases, G protein-coupled receptors, and growth factor receptors [37]. Therefore, Src inhibitors are used *in* *vivo* and exhibit the ability to suppress the pulmonary inflammation [38]. Importantly, Src inhibition may provide attractive target in the treatment of devastating ALI. In this study, we found high V_T_ ventilation could induce Src activation by phosphorylation to a plateau during the first 4 hours and hyperoxia further enhanced the activation of Src. Notably, iPSCs can suppress the activation of Src simulating the inhibitory effect provided by knockout of Src in mice.

Oxidants can modify cellular lipids, protein, and DNA to cause their aberrant function due to peroxidation. Increased ROS production in response to mechanical stretch of lung has been widely delineated in VILI [39], [40]. The NADPH oxidase, a membrane-bound multi-component enzyme complex, has been implicated as major source for increased superoxide (·O_2_^−^) production respond to mechanical stress in pulmonary epithelial cells [21]. Excessive generation of ROS by NADPH oxidase is commonly thought to be responsible for tissue injury associated with ventilator-associated lung injury and ARDS [41]. In this study, we demonstrated hyperoxia augmented stretch-induced NADPH oxidase activity and the concentration of MDA, an aldehydic secondary product of lipid peroxidation used as a marker of oxidative damage. Importantly, iPSCs was shown to suppress the activity of NADPH oxidase and MDA formation simulating the inhibitory effect by knockout of Src in mice. These results suggest Src is upstream to NADPH oxidase signaling in our hyperoxia-augmented VILI model. Consistent with our results, another study by Chowdhury et al. demonstrated that *in* *vitro* hyperoxia activated Src and activation of Src regulated NADPH oxidase-mediated ·O_2_^−^ production via p47*^phox^* tyrosine phosphorylation in lung endothelial cells. Inhibition of Src activation by specific inhibitor prevented hyperoxia-mediated phosphorylation of Src, translocation of p47*^phox^* to the cell periphery, and generation of ROS mediated by NADPH oxidase [24].

Further investigation was conducted of the upregulation of NADPH oxidase by Src activation and then produced large amount of oxidative stress in the setting of hyperoxia-augmented VILI. According to previous results by Chiang et al., high V_T_ ventilation stimulated the production of ROS, which was mediated by NADPH oxidases (NOXs) and apocynin can attenuate VILI as the NADPH oxidase inhibitor [42]. However, the major ROS generating enzyme NOX is present in phagocytes as well as non-phagocytic cells. A number of homologs of NOX in mammalian have been discovered, comprising NOX1-5 and two larger Dual Oxidases, DUOX1 and DUOX2, which are widely expressed in many cell types to mediate the biological responses, such as cell mitosis, differentiation, migration, and immune regulation. NOX enzymes are also involved in a variety of pathologies of respiratory diseases, including acute lung injury, emphysema, and pulmonary fibrosis [41]. NOX1 has been shown to play a crucial role in hyperoxia-induced lung injury in mice exposed to 100% O_2_ for 72 hours [43]. In particular, NOX2 has been established to be a major source of ROS associated with pulmonary inflammation. However, no precise investigation of NOX1 and NOX2 in the lungs has been studied in the setting of acute hyperoxia-augmented VILI. In this study, we found high V_T_ ventilation increased both NOX1 and NOX2 but hyperoxia further induced the increase of NOX2. Although the levels of both NOX1 and NOX2 were decreased in Src-KO mice, the level of NOX2 was significantly inhibited to a greater degree than that of NOX1 (Figures 4A, 4B). Most interestingly, iPSCs can suppress the production of NOX2, not NOX1, simulating the inhibitory effect on NOX2 exerted by knock-out of Src in mice (Figures 4A, 4B). These results implicate that Src activation is involved in the membrane-bound NOX2 and intracellular ROS generation in our animal model. Src is an important upstream kinase that regulates NADPH oxidase-induced ROS formation and iPSCs can inhibit the activation of Src and downstream NOX2-derived ROS.

The results showed that high V_T_ ventilation concomitant with hyperoxia further recruited the influx of neutrophils as measured by infiltrating neutrophils of BAL fluid and total neutrophil sequestration by MPO levels of lungs (Figures 3A, 3B) and increased the production of MIP-2 and PAI-1 (Figures 3C, 3D) and epithelial apoptosis by ultrastructural image and TUNEL staining (Figures 6A, 6B, 6C). Neutrophils attracted by MIP-2, a potent chemokine, are the major inflammatory cells to generate ROS associated with ALI [40]. PAI-1 can inhibit both urokinase-type plasminogen activator (uPA) and tissue-type plasminogen activator (tPA). Notably, patients with ALI supported by high V_T_ ventilation had increased local production of PAI-1 in BAL, leading to suppress the fibrinolytic activity and form the fibrin [44]. Moreover, PAI-1 knockout mice were proved to be protected from acute hyperoxic lung injury [45]. In this study, we demonstrated iPSCs can attenuate the acute pulmonary inflammation and coagulation cascades by reducing the neutrophil trafficking into the lung and decreasing both proinflammatory cytokine MIP-2 and anti-fibrinolytic mediator PAI-1. Otherwise, iPSCs was also shown to decrease the alveolar epithelial apoptosis measured by TUNEL staining and TEM image and restore the histological architecture and improve the oxygenation evaluated by decreased alveolar-arterial oxygen difference (A-aDO2). Taken together, iPSCs can suppress these acute pulmonary inflammation and epithelial apoptosis through the inhibition of Src activation simulating the repressive effect in Src knockout mice. In fact, emerging evidences indicate that Src plays an important role for intracellular signaling transduction for acute pulmonary inflammation and apoptotic cell death [22]–[24]. Thus, iPSCs possess the anti-inflammatory and anti-apoptotic abilities attributing to suppressing the Src-dependent signaling.

Stem cell therapy has been considered to be a potential therapy to treat ALI. In addition to their cell-to-cell contact-dependent differentiation into multipotent alveolar progenitors, iPSCs may modulate the pathophysiological process of lung diseases through cell-contact independent paracrine effects [6], [32]. Moreover, oxidant stress is known to mediate inflammation and activation of NF-κB and activator protein-1 (AP-1) [12]. These redox-sensitive transcription factors can be activated for the production of major proinflammatory cytokines in VILI and hyperoxia [40], [41]. In our previous study, we have shown that iPSCs can suppress the activity of neutrophils to secrete MIP-2 by LPS stimulation in a cell-contact independent manner [32]. Additionally, we demonstrated that iPSC therapy can attenuate the severity of VILI in mice in vivo despite lower percentage of stem cells engrafted into the lung and the conditioned medium of iPSCs had beneficial effects similar to those of iPSCs [6]. In the present study, we have demonstrated herein iPSCs could reduce the high V_T_ ventilation and concomitant hyperoxia-induced activation of Src, NADPH oxidase and subsequently block oxidant-responsive inflammatory signaling, thus mitigating the hyperoxia-augmented VILI. Because there are only less than 4% of iPSCs trafficked in the injured lungs of mice after 4 h of mechanical ventilation, the beneficial effects of cell therapy in the restoration of lung damage in a short duration are due to both the effects of iPSCs and their soluble factors in the conditioned medium [6], [32]. In consistent with our results, Wen et al. reported intravenous administration of amniotic fluid stem cells reduced the hyperoxia-induced pulmonary inflammation and early-stage fibrosis in a mouse model of hyperoxia-induced ALI [46]. Chang et al. demonstrated that intratracheal administration of umbilical cord blood-derived MSCs attenuated hyperoxia-induced lung injury in neonatal rats through suppressing both cytosolic and membrane p47^phox^ [25]. However, the precise molecular mechanisms were not shown in the above two studies. In our results, we have demonstrated iPSCs exerted the anti-oxidant, anti-inflammatory, and anti-apoptotic abilities to reduce the acute hyperoxic VILI. Through inhibiting the activation of Src, NOX2, and NADPH oxidase activity, iPSCs can attenuate oxidative stress and subsequent inflammatory responses and apoptotic cell death. Although iPSC therapy has been shown to suppress the hyperoxia-augmented VILI via Src signaling in part, further studies would be investigated to unravel other mechanistic pathways.

Given the high risk of tumorigenicity following iPSCs transplantation, we have made efforts to refine our iPSCs procedure to remove oncogene by activating Poly (ADP-ribose) polymerase 1 (Parp1) to replace Klf4 or c-Myc [35]. Parp1, a highly conserved DNA-binding protein and abundant in the nucleus, would regulate DNA methylation, chromosome structure, and transcription. Parp1 functions mainly as forming ADP-ribose group on its target protein, and which is called PARylation. Importantly, Parp1 is the major protein that catalyzes PARylation in their family. Sox2, a pivotal transcription factor in reprogramming, could be PARylated and facilitate the efficient iPSC generation through this post-translational modification. In reprogramming, somatic cells suffer greater stress caused by chromosome remodeling. However, Parp1 would regulate the p53 that promotes cell survival by PARylation in stress situation. In this study, we used Oct4/Sox2/Parp1-reprogrammed iPSCs to avoid the ethical controversy and possibility of oncogenic factor-induced tumorgenicity and improve the efficiency of iPSC generation and cell survival. These efforts would advance the iPSC therapy to the clinical use for personalized medicine.

## Conclusions

In patients with severe ARDS, using MV with high levels of oxygen during the first few hours after intubation is necessary. By using an *in* *vivo* mouse hyperoxia-augmented VILI model, we demonstrated that high V_T_ ventilation and concomitant hyperoxia can induce lung injury associated with neutrophil influx, oxidative stress, alveolar epithelial-capillary damage, and production of MIP-2 and PAI-1. Hyperoxia further aggravated high V_T_-induced ALI. Severe inflammation, edema, pathologic destruction, and impaired gas exchange of injured lungs were attenuated by OSP-iPSCs and were, at least partially, mediated by inhibiting the Src-dependent NOX2-ROS pathway. Notably, iPSC therapy revealed potent anti-oxidant, anti-inflammatory and anti-apoptotic abilities to counteract combined deleterious effects in our animal model of hyperoxia-augmented VILI. Understanding the molecular basis of iPSCs related to the suppression of the Src-NOX2-ROS signaling pathway, pulmonary inflammation, and apoptosis may allow clarification of the pathophysiological mechanisms regulating VILI and hyperoxia and provide insight to develop novel therapeutic treatments for ARDS.

## Materials and Methods

### Ethics of experimental animals

We obtained male C57BL/6, either wild-type or Src-deficient on a C57BL/6 background, weighing between 20 and 25 g, aged between 2 and 3 months, were obtained from Jackson Laboratories (Bar Harbor, ME) and National Laboratory Animal Center (Taipei, Taiwan) [47]. Briefly, heterozygotes (Src^+/−^) were used because mice homozygous for Src^tm1Sor^ targeted mutation (Src^−/−^) exhibit growth retardation, failure of tooth eruption, osteopetrosis with lack of secondary bone resorption, and lethality at 3–4 weeks [47], [48]. Mice that were heterozygous for the Src^tm1Sor^ mutation (Src^+/−^); however, have no apparent abnormalities [47], [48]. The target mutation of Src was constructed by inserting a neomycin cassette into the first coding exon and is electroporated into 129S7/SvEvBrd-Hprt derived AB2.1 embryonic stem (ES) cells [47], [48]. Chimeras were generated through injecting these ES cells into C57BL/6 (B6) blastocysts. The resulting chimeric male animals are crossed to wild-type C57BL/6 mice, and then backcrossed to the same for 10 generations. The lower expressions of the Src protein in Src^+/−^ mice were confirmed by using Western blot analysis. We followed the recommendations in the Guide for the Care and Use of Laboratory Animals of the NIH to perform the study. The Institutional Animal Care and Use Committee of Chang Gung Memorial Hospital approved the protocol (Permit number: 2011093005). All surgery was conducted under ketamine and xylazine anesthesia to minimize suffering of animals.

### Experimental groups

Animals were randomly distributed into 6 groups in each experiment: group 1, control, nonventilated wild-type mice with room air (n = 5 for Western blot, Evans blue dye (EBD) assay, lung water, cell counts, myeloperoxidase (MPO), malondialdehyde (MDA), NADPH oxidase assay, histology, immunohistochemistry, terminal deoxynucleotidyl transferase-mediated dUTP-biotin nick end-labeling (TUNEL) assay, electron microscopy, and MIP-2 and PAI-1); group 2, control, nonventilated wild-type mice with hyperoxia (n = 5 for Western blot, EBD assay, cell counts, MPO, MDA, NADPH oxidase assay, histology, immunohistochemistry, TUNEL assay, electron microscopy, and MIP-2 and PAI-1); group 3, V_T_ 30 mL/kg wild-type mice with room air (n = 5 for Western blot: 60 min, 120 min, and 240 min, EBD assay, cell counts, MPO, MDA, NADPH oxidase assay, histology, immunohistochemistry, TUNEL assay, electron microscopy, and MIP-2 and PAI-1); group 4, V_T_ 30 mL/kg wild-type mice with hyperoxia (n = 5 for Western blot: 60 min, 120 min, and 240 min, EBD assay, cell counts, MPO, MDA, NADPH oxidase assay, histology, TUNEL assay, immunohistochemistry, electron microscopy, and MIP-2 and PAI-1); group 5, V_T_ 30 mL/kg wild-type mice after iPSCs administration with hyperoxia (n = 5 for Western blot, EBD assay, cell counts, MPO, MDA, NADPH oxidase assay, histology, immunohistochemistry, TUNEL assay, electron microscopy, and MIP-2 and PAI-1); group 6, V_T_ 30 mL/kg Src^+/−^ mice with hyperoxia (n = 5 for Western blot, EBD assay, cell counts, MPO, MDA, NADPH oxidase assay, histology, immunohistochemistry, TUNEL assay, electron microscopy, and MIP-2 and PAI-1).

### Ventilator protocol

We used our previously established mouse model of VILI [19]. One hour of MV was employed for Western blot analysis, and 4 h was applied for MIP-2 and PAI-1 production, cell counts, lung water, Evans blue dye, myeloperoxidase, free radicals, electron microscopy, and histologic staining analyses, based on our time-course and previous studies [7], [19].

### NADPH oxidase assay

The lungs were homogenized in NADP or NADPH extraction buffer. The NADP^+^ and NADPH in the protein extracts were measured using the EnzyChrom NADP^+^/NADPH assay kit (BioAssay Systems, Hayward, CA) based on a glucose dehydrogenase cycling reaction, in which the formed NADPH reduces a formazan (MTT) reagent. Each sample was run in duplicate and expressed as NADP^+^/NADPH ratio according to the manufacturer’s instructions.

### Statistical evaluation

We quantitated Western blot analysis using a NIH image analyzer Image J 1.27z (National Institutes of Health, Bethesda, MD) and the results were presented as arbitrary units. The data were expressed as the mean ± SD from at least 5 experiments. The data of EBD assay, lung wet-to-dry weight ratio, MIP-2 and PAI-1 MDA, NADP^+^/NADPH, histopathologic assay, and oxygenation were analyzed using Statview 5.0 (Abascus Concepts Inc. Cary, NC; SAS Institute, Inc.). The results of Western blots were normalized to the nonventilated control wild-type mice with room air. We employed ANOVA to assess the statistical significance of the differences, followed by multiple comparisons with a Scheffe’s test, and a P value less than 0.05 was considered statistically significant.

Ventilator protocol, immunoblot analysis, immunohistochemistry, histopathologic grading of VILI, terminal deoxynucleotidyl transferase-mediated dUTP-biotin nick end-labeling assay, generation of iPSC lines and cell culture, nuclear reprogramming, ALP, SSEA1 staining, analysis of lung water, cell counts, EBD analysis, MPO assay, transmission, electron microscopy and measurements of malondialdehyde, MIP-2, and PAI-1 were performed as previously described [7], [18], [19], [35], [49].
